# Supplementary material for: Convergent evolution of linked mating-type loci in basidiomycete fungi
Source: PLoS Genet. 2019 Sep 6;15(9):e1008365. doi: 10.1371/journal.pgen.1008365 (PMC6730849; doi:10.1371/journal.pgen.1008365)
Supplement: S1 Fig — A. Genotyping of the V. humicola isolates with modified RAPD method. Stars highlight the isolates that show identical genotyping profiles as that of the V. humicola type strain, CBS571, and have ITS sequences that classify them as V. humicola. B. k-mer frequency distribution for V. humicola CBS4282 Illumina reads. A k-mer length of k = 31 was used for the analysis. A single main peak can be observed. The rise in k-mer occurrence below a k-mer frequency of 10 is due to sequencing errors. The sum of k-mers from the main peak results in an estimate of 22.6 Mb for the size of the haploid genome. k-mer frequency analysis was performed as described [122, 123]. C. Genome comparison between V. humicola strains JCM1457 (A2) and CBS4282 (A1). Regions of sequence similarity were determined by nucmer and plotted with Circos. Sizes are given in Mb. The positions of the MAT regions are indicated as grey bars. (PDF) [file pgen.1008365.s001.pdf]

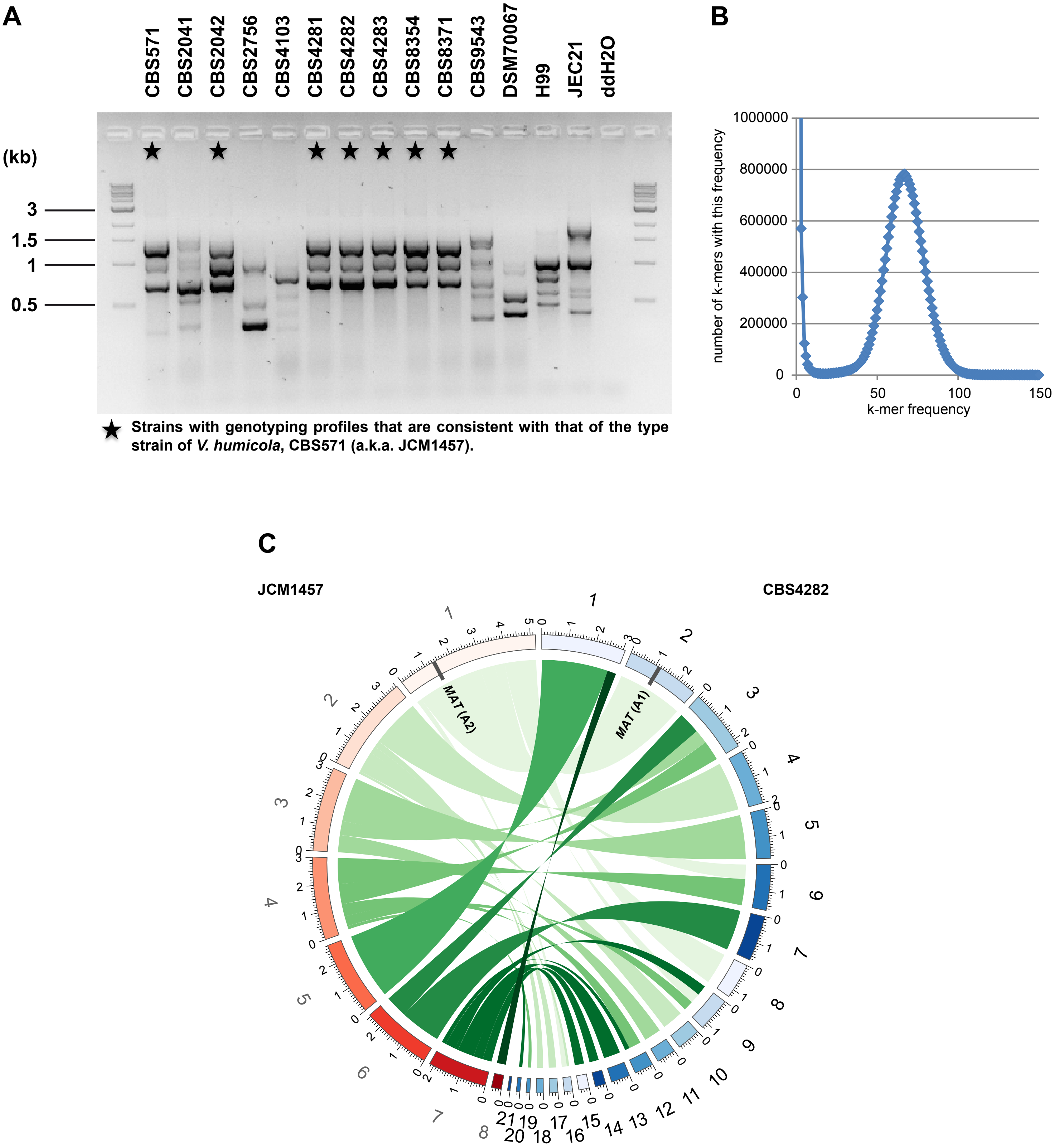
A
CBS571
CBS2041
CBS2042
CBS2756
CBS4103
CBS4281
CBS4282
CBS4283
CBS8354
CBS8371
CBS9543
DSM70067
H99
JEC21
ddH2O
(kb)
3
1.5
1
0.5
★ Strains with genotyping profiles that are consistent with that of the type strain of V. humicola, CBS571 (a.k.a. JCM1457).
B
number of k-mers with this frequency
1000000
800000
600000
400000
200000
0
0
50
100
150
k-mer frequency
C
JCM1457
CBS4282
MAT (A2)
MAT (A1)
