# Supplementary figures and images for: Convergent evolution of linked mating-type loci in basidiomycete fungi
Source: PLoS Genet. 2019 Sep 6;15(9):e1008365. doi: 10.1371/journal.pgen.1008365 (PMC6730849; doi:10.1371/journal.pgen.1008365)

**A**

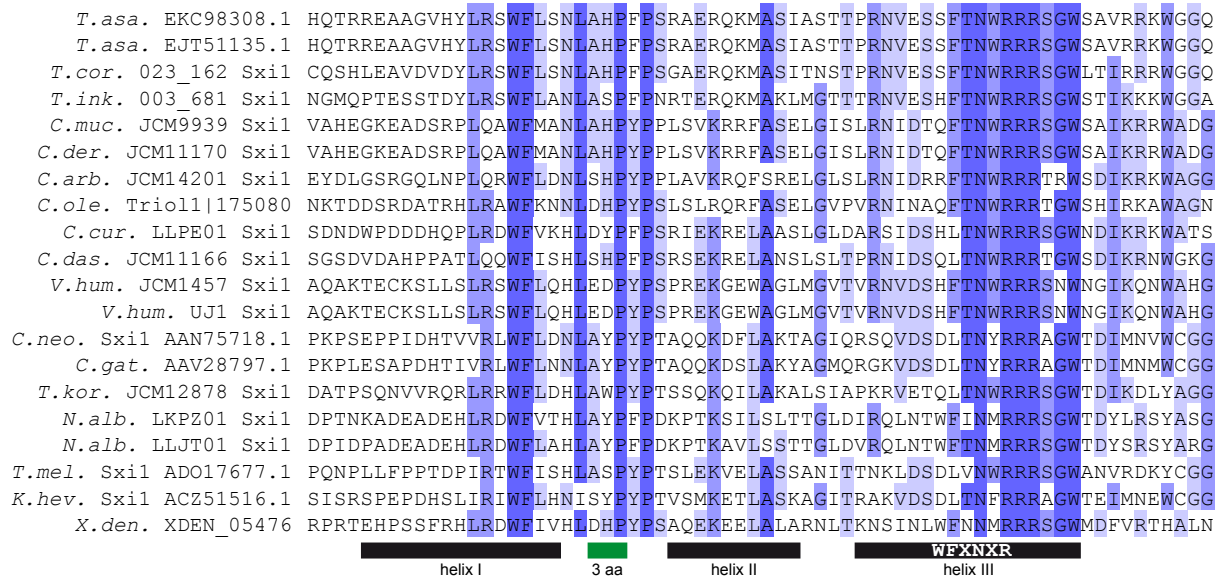

**B**

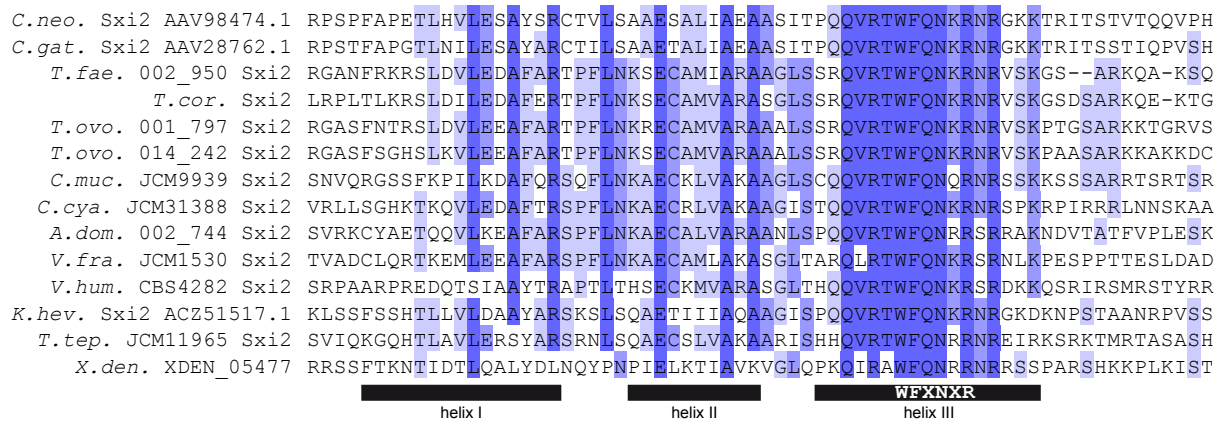

Supplement: S2 Fig — A. Multiple alignment of the homeodomains from Sxi1 homologs (class HD1 homeodomain transcription factors) from Tremellomycetes. The three conserved helices are underlined in black. A three amino acid insertion characteristic for the HD1 homeodomain factors (in contrast to the HD2 homeodomains) is underlined in green. The conserved DNA binding motif WFXNXR within helix III is indicated. Labelling of helices and motifs according to [16]. Species: C.neo. Cryptococcus neoformans, C.gat. Cryptococcus gattii, C. arb. Cutaneotrichosporon arbiformis, C.cur. Cutaneotrichosporon curvatus, C. das Cutaneotrichosporon daszweskae, C. der Cutaneotrichosporon dermatis, C. muc. Cutaneotrichosporon mucoides, C.ole. Cutaneotrichosporon oleaginosum, K.hev. Kwoniella heveanensis, N.alb. Naganishia albida, T. kor. Takashimella koratensis, T.asa. Trichosporon asahii, T.cor. Trichosporon coremiiforme, T.ink. Trichosporon inkin, T.mel. Tremella mesenterica, V.hum. Vanrija humicola, X.den. Xanthophyllomyces dendrorhous. B. Multiple alignment of the homeodomains from Sxi2 homologs (class HD2 homeodomain transcription factors) from Tremellomycetes. Labelling and species names as in A with addition of the following species: A.dom. Apiotrichum domesticum, C. cya. Cutaneotrichosporon cyanovorans, T. tep. Takashimella tepidaria, T.fae. Trichosporon faecale, T.ovo. Trichosporon ovoides, V. fra. Vanrija fragicola. (PDF) [file pgen.1008365.s002.pdf]

A

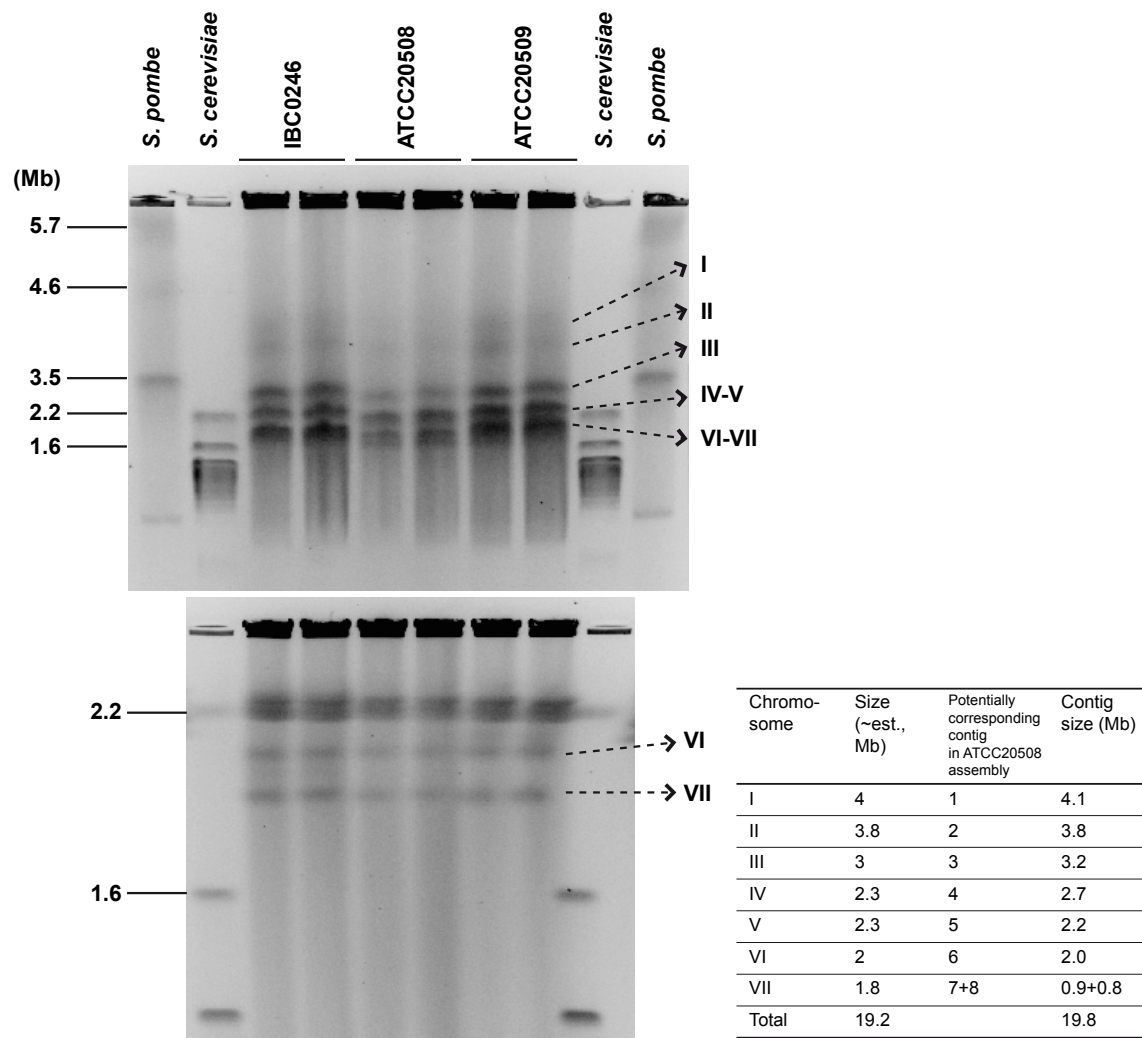

B

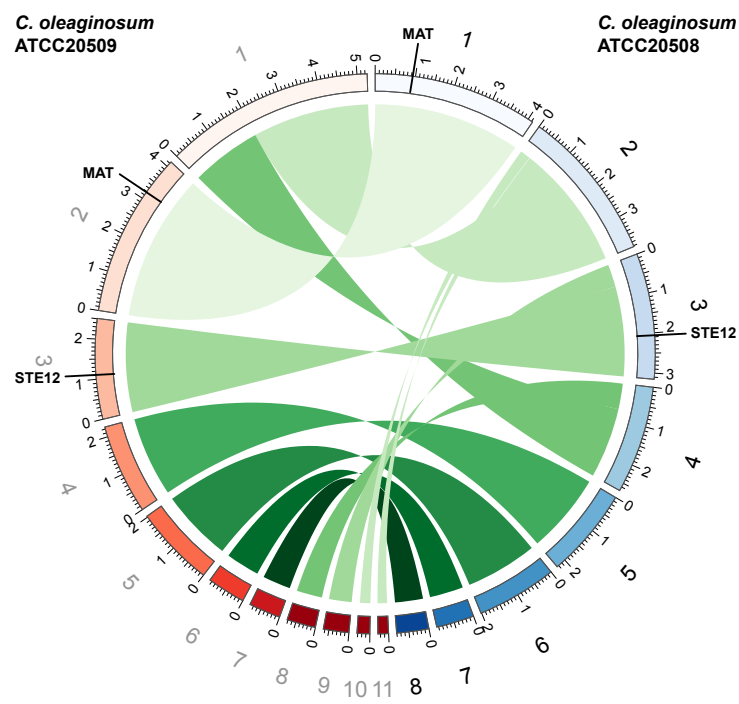

Supplement: S4 Fig — The genome was sequenced with Pacific Biosciences SMRT sequencing and assembled into eight contigs with 8208 predicted genes. A. CHEF analysis of the three C. oleaginosum isolates. The image on top shows separation of the larger chromosomes, with S. pombe and S. cerevisiae serving as markers; the image at the bottom shows separation of the smaller chromosomes of the same three C. oleaginosum isolates, with only S. cerevisiae included as marker (please see Materials and Methods for details). Two plugs prepared from independent cultures were included for each isolate. The table summarizes the estimated sizes of the seven chromosomes identified by the CHEF analysis. The sizes of the six largest chromosomes correspond well to the sizes of the six largest contigs of the ATCC20508 assembly. Similar to the other two strains, ATCC20508 carries the MAT A2 allele and STE12 on different contigs (contigs 1 and 3, respectively, see part B), making it unlikely that they are located on the same chromosome. B. Genome comparison between C. oleaginosum strains ATCC20508 (A2) and ATCC20509 (A2). Regions of sequence similarity were determined by nucmer and plotted with Circos. Sizes are given in Mb. The positions of the MAT regions and STE12 are indicated. (PDF) [file pgen.1008365.s004.pdf]

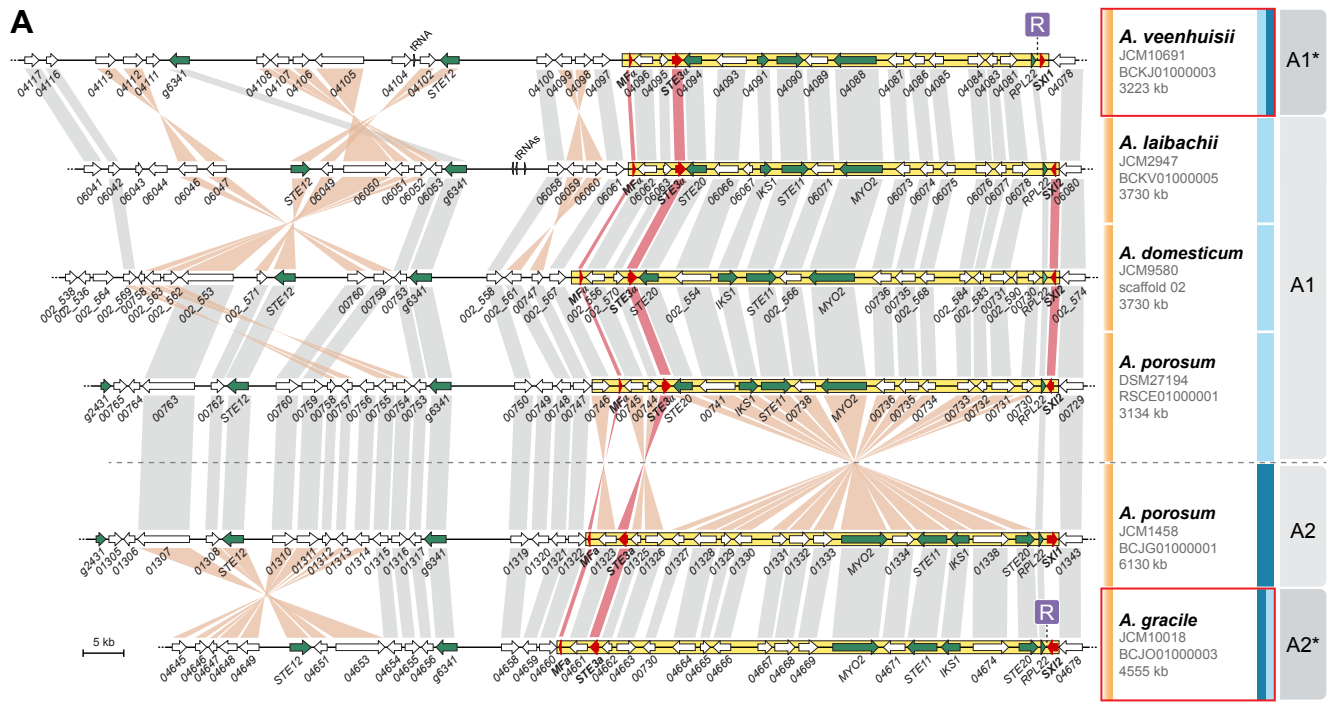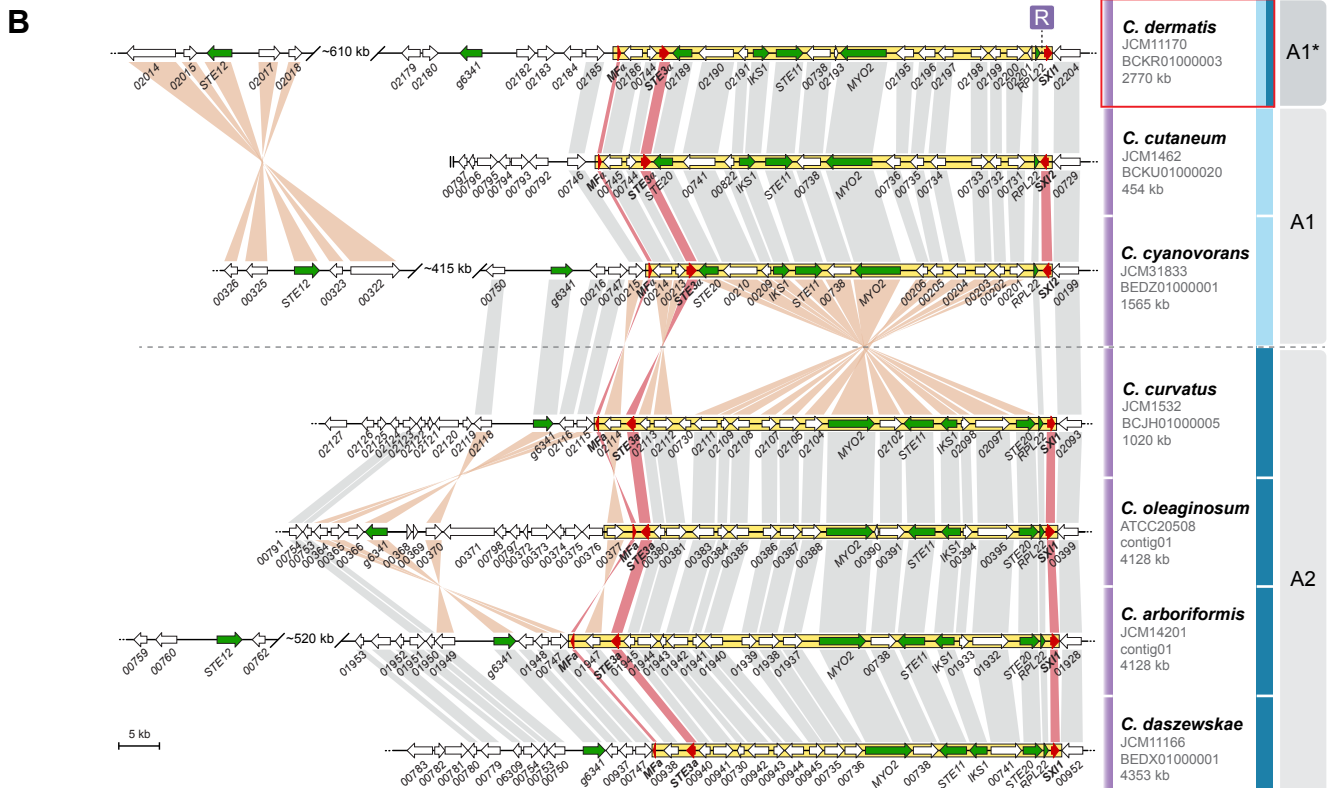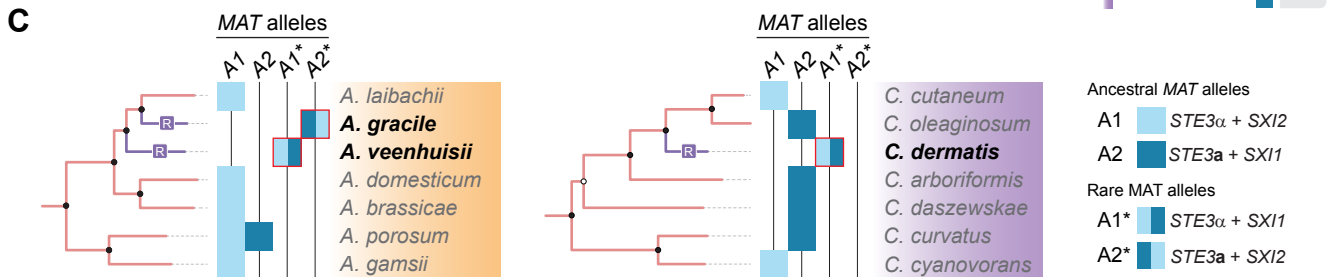

Supplement: S5 Fig — MAT loci of Apiotrichum (A) and Cutaneotrichosporon (B) species. Classic mating type-defining genes (SXI1 or SXI2, STE3, and the pheromone precursor genes) are shown in red, other genes that are part of the mating type locus or flanking the mating type locus (gene g6341) of C. neoformans are shown in green, and genes not present in the C. neoformans mating type locus are shown in white. The MAT allele is indicated on the right, with A1/A1* and A2/A2* MAT alleles shown above and below the dashed line, respectively. The proposed MAT locus region is enclosed in a yellow box in each strain. Orthologs are connected by grey or orange bars when in the same or opposite orientations, respectively. Strains carrying the A1* or A2* alleles are outlined in red. Predicted recent recombination events leading to the A1* and A2* alleles are indicated by the letter R. The MAT locus of the hybrid species C. mucoides is shown in S6 Fig together with MAT loci from other hybrid species. C. Phylogenetic trees showing species relationships within the genera Apiotrichum and Cutaneotrichosporon. The MAT alleles of the strains are indicated, and predicted recent recombination events leading to the A1* and A2* alleles are indicated by the letter R at the corresponding branches of the phylogenetic trees. (PDF) [file pgen.1008365.s005.pdf]

A

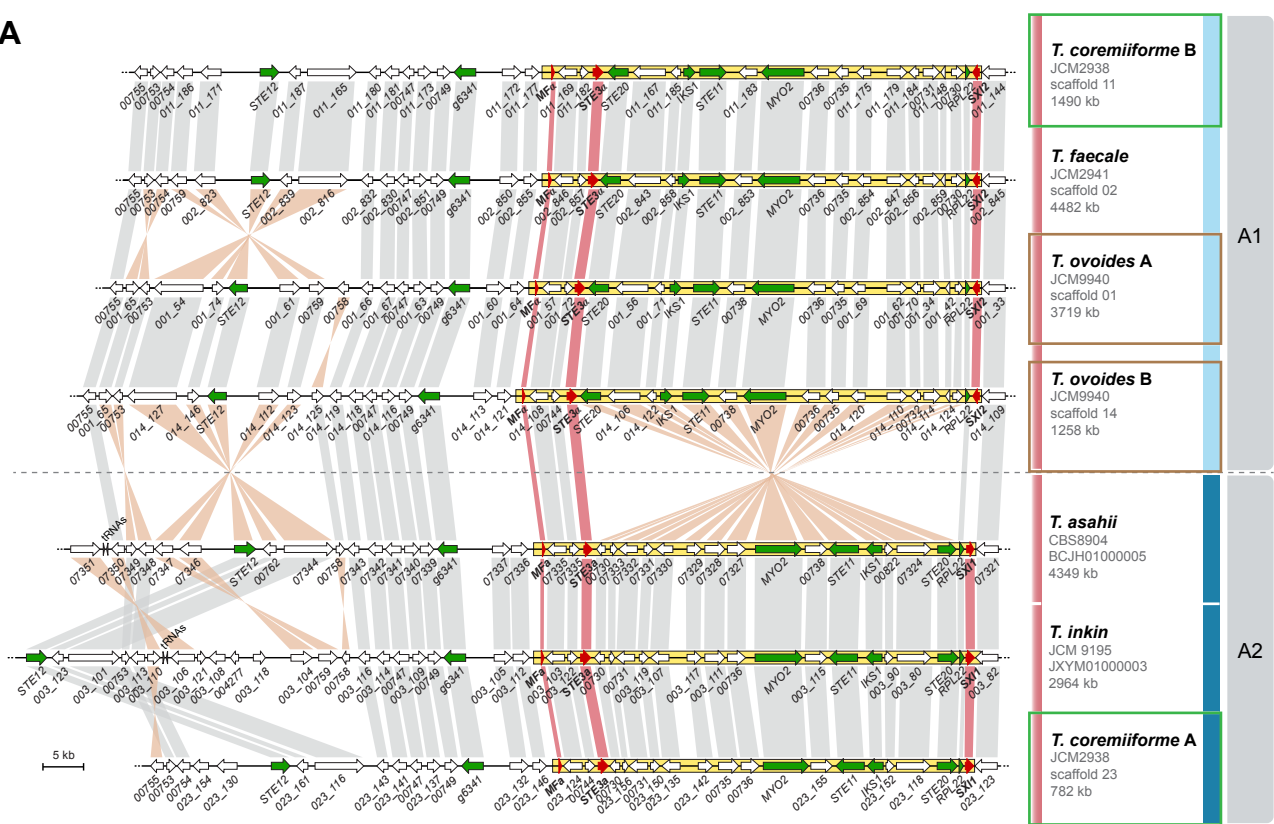

B

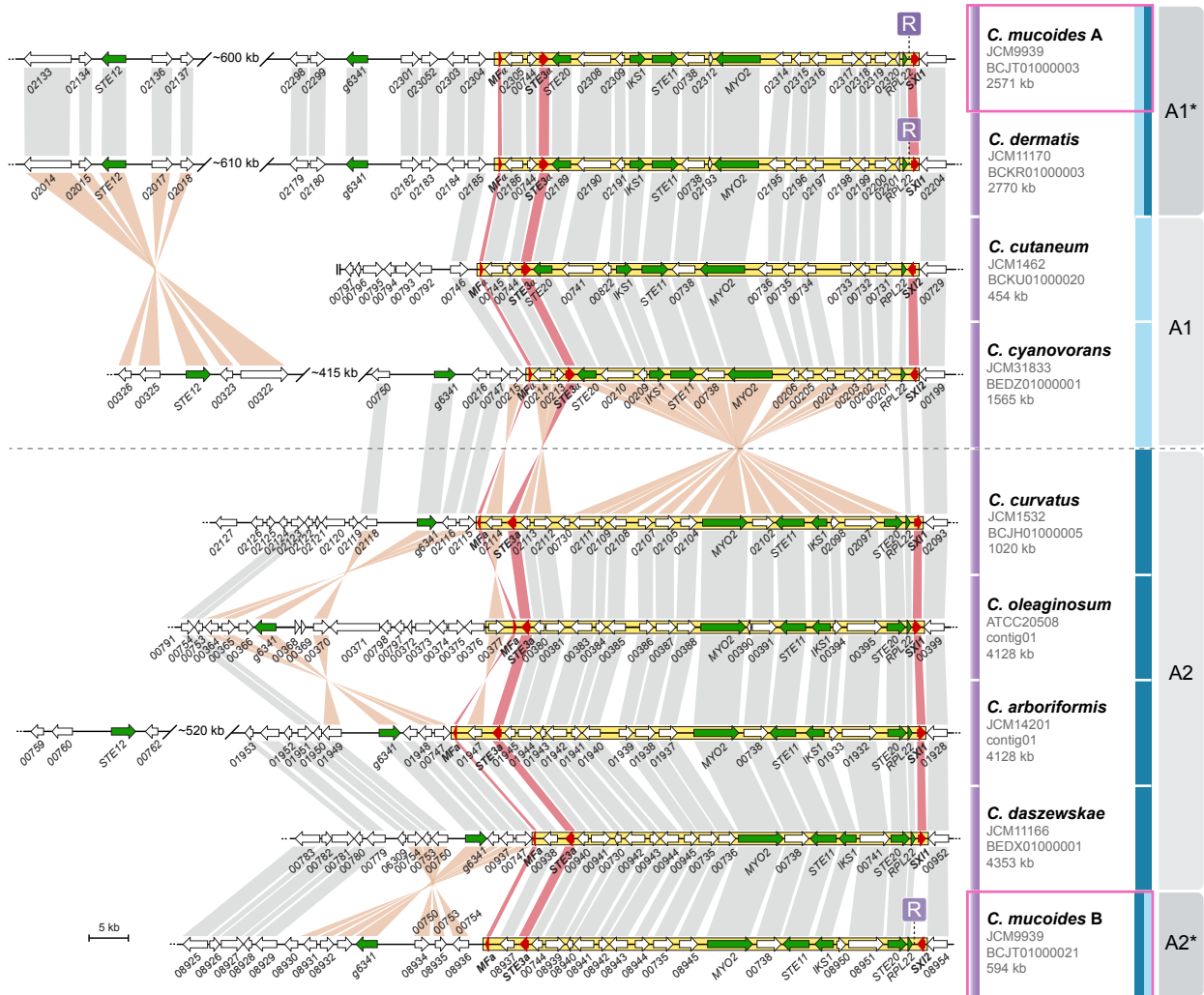

C

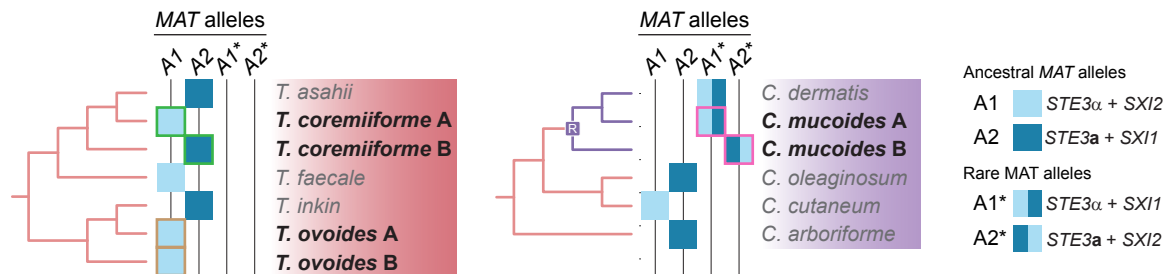

Supplement: S6 Fig — MAT loci of Trichosporonales lineages containing hybrid species (Trichosporon species in A, Cutaneotrichosporon species in B). Classic mating type-defining genes (SXI1 or SXI2, STE3, and the pheromone precursor genes) are shown in red, other genes that are part of the mating type locus or flanking the mating type locus (gene g6341) of C. neoformans are shown in green, and genes not present in the C. neoformans mating type locus are shown in white. The MAT allele is indicated on the right, with A1/A1* and A2/A2* MAT alleles shown above and below the dashed line, respectively. The proposed MAT locus region is enclosed in a yellow box in each strain. Orthologs are connected by grey or orange bars when in the same or opposite orientations, respectively. Predicted recent recombination events leading to the A1* and A2* alleles are indicated by the letter R. MAT alleles of hybrid strains are outlined by colored boxes around the strain names on the right. C. Phylogenetic trees showing species relationships within the genera Trichosporon and Cutaneotrichosporon. The MAT alleles of the strains are indicated, and a predicted recent recombination event leading to the A1* and A2* alleles in Cutaneotrichosporon is indicated by the letter R at the corresponding branch of the phylogenetic tree. Names of hybrid species are shown in bold. Each hybrid species carries two subgenomes designated A and B, with homeologs for each gene still present in both subgenomes [28, 52]. For more information about the hybrid strains, please see S2 Text. (PDF) [file pgen.1008365.s006.pdf]

Key:

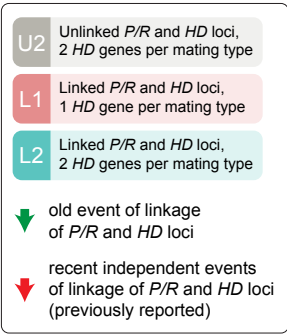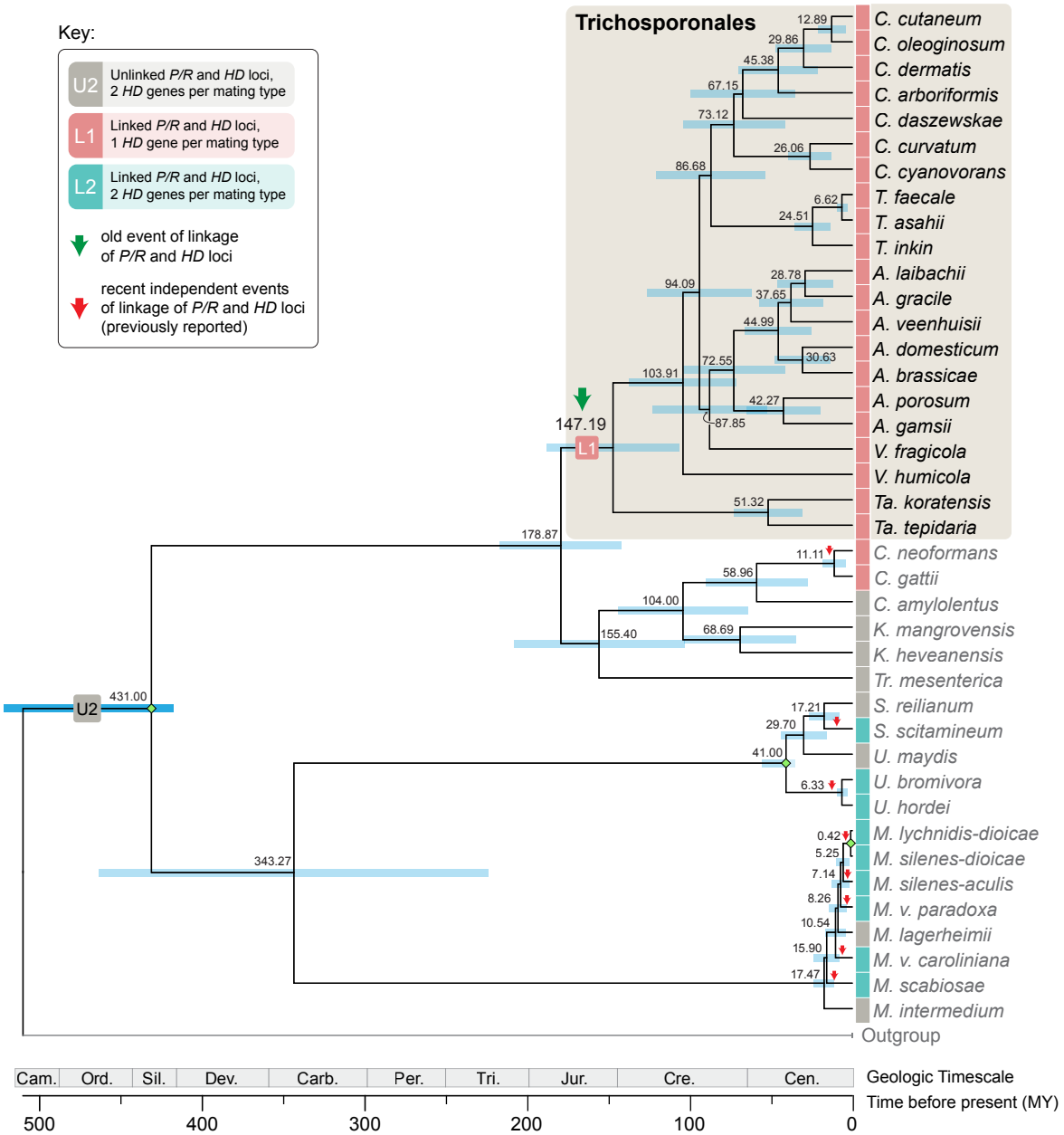

Supplement: S7 Fig — The reconstructed species tree shown in Fig 1, with branch lengths in the units of number of substitutions per site, was used as input and transformed into an ultrametric tree with relative times. The final timetree was obtained by converting the relative node ages into absolute dates by using three calibration constraints: 0.42 million year (MY) corresponding to the divergence between Microbotryum lychnidis-dioicae and Microbotryum silenes-dioicae [141]; 41 MY for the Ustilago—Sporisorium split; and 413 MY representing the minimum age of Basidiomycota. The latter two calibration points were obtained from the Timetree website (http://www.timetree.org/), which should be referred to for additional information and references. Numbers on tree nodes indicate the inferred dates of speciation. Events of mating-type loci linkage are indicated by red or green arrows (see key). The blue bars correspond to 95% confidence intervals. (PDF) [file pgen.1008365.s007.pdf]

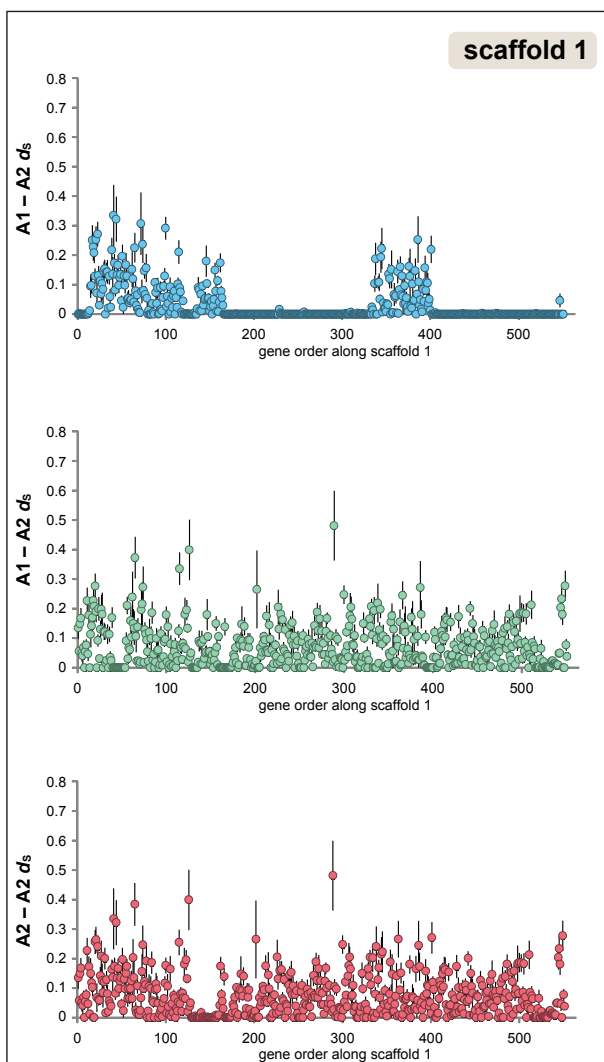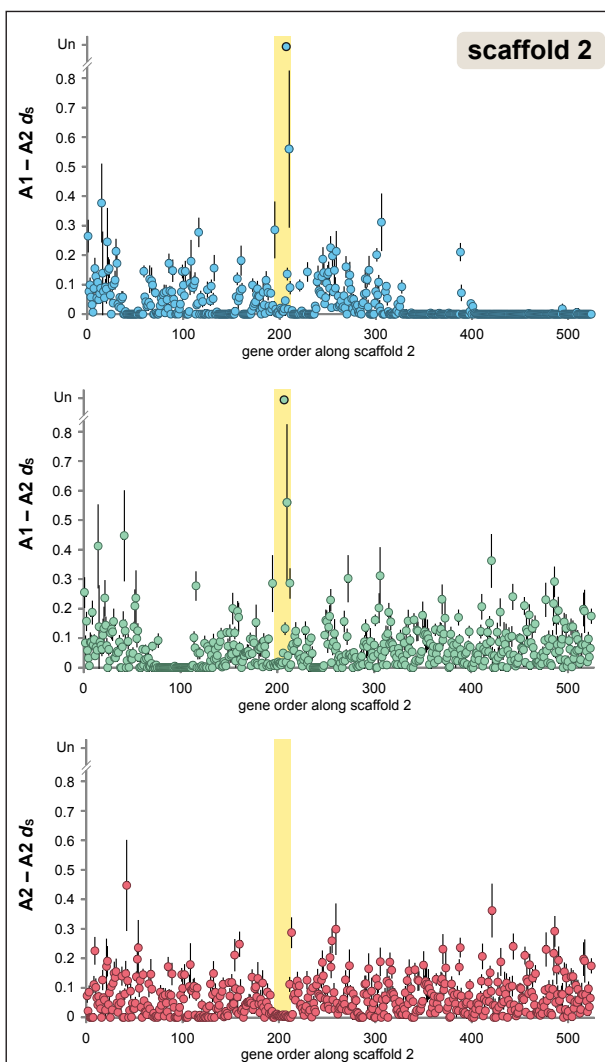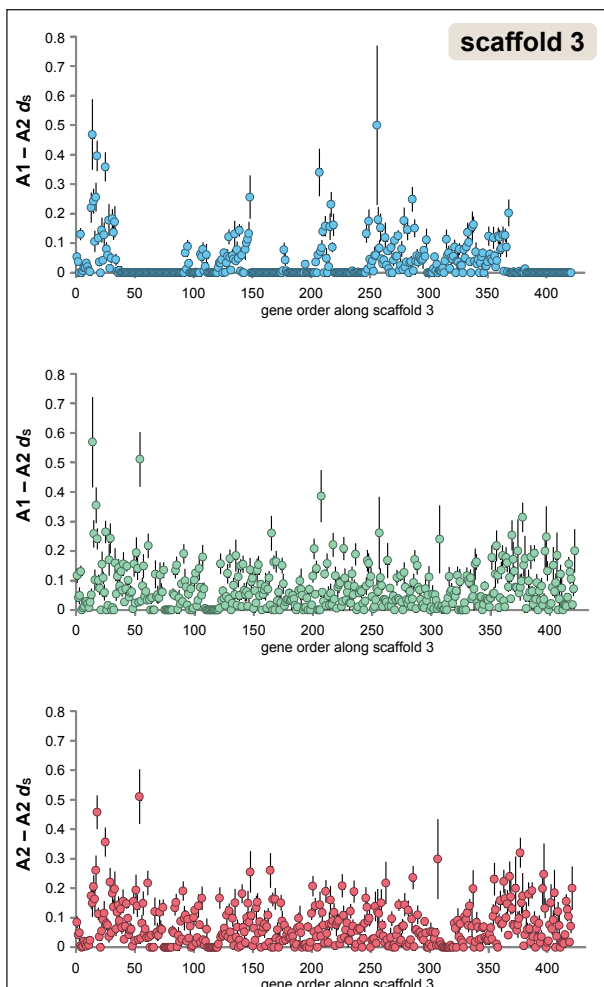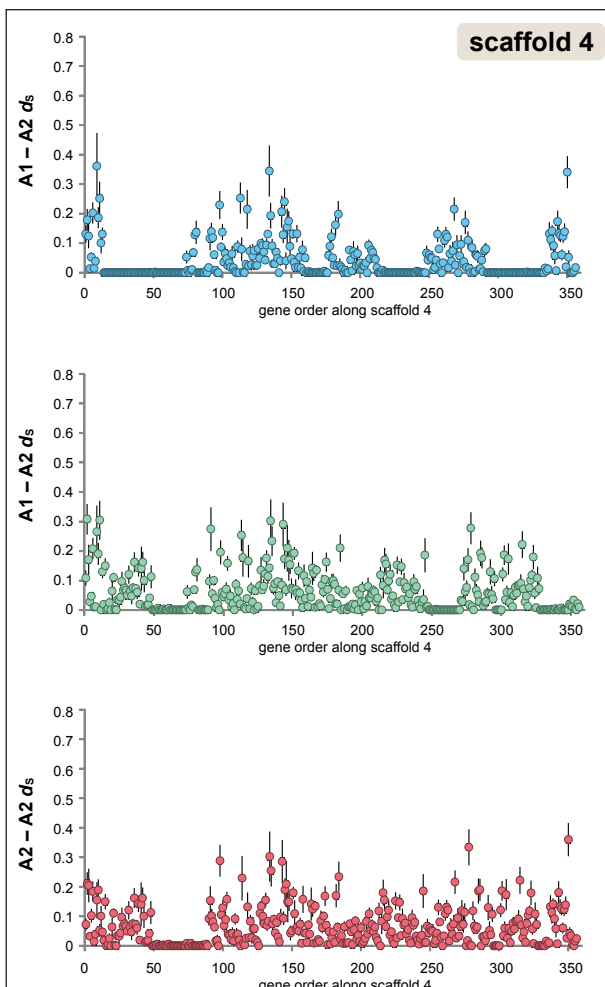

● CBS4282 (A1) vs. JCM1457 (A2) ● CBS4282 (A1) vs. UJ1 (A2) ● JCM1457 (A2) vs. UJ1 (A2)

Supplement: S8 Fig — Synonymous substitutions per synonymous site and standard errors (dS +/- SE) are shown for pairwise comparisons between strains CBS4282, JCM1457, and UJ1 for genes on the four longest scaffolds (scaffold 2 contains the MAT locus) of strain CBS4282. Only genes for which a dS value could be calculated are shown. For the STE3 genes, no dS value could be calculated in comparisons of strains with different mating types, and it is represented by Un (undetermined). The location of the core MAT region (between the HD and P/R genes) on scaffold 2 of CBS4282 is labelled in yellow. (PDF) [file pgen.1008365.s008.pdf]

**A**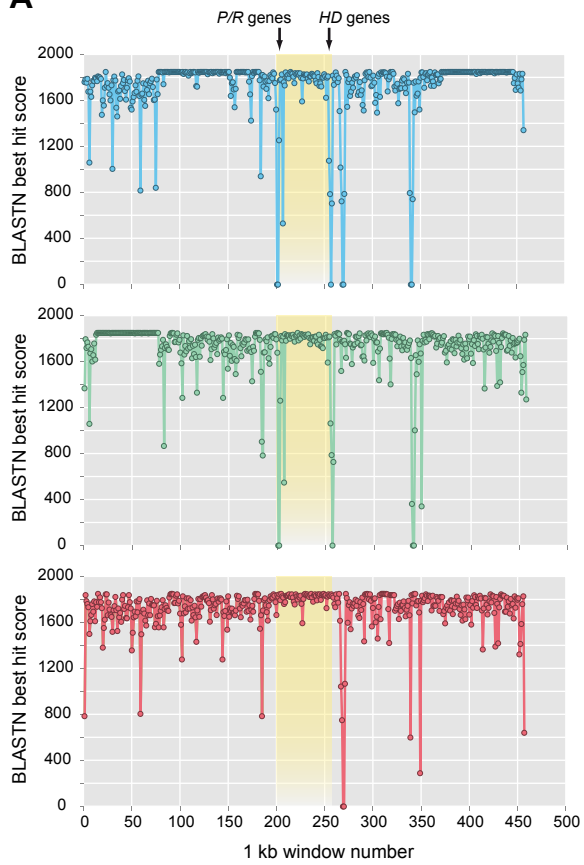

- CBS4282 (A1) vs. JCM1457 (A2)
- CBS4282 (A1) vs. UJ1 (A2)
- JCM1457 (A2) vs. UJ1 (A2)

**B**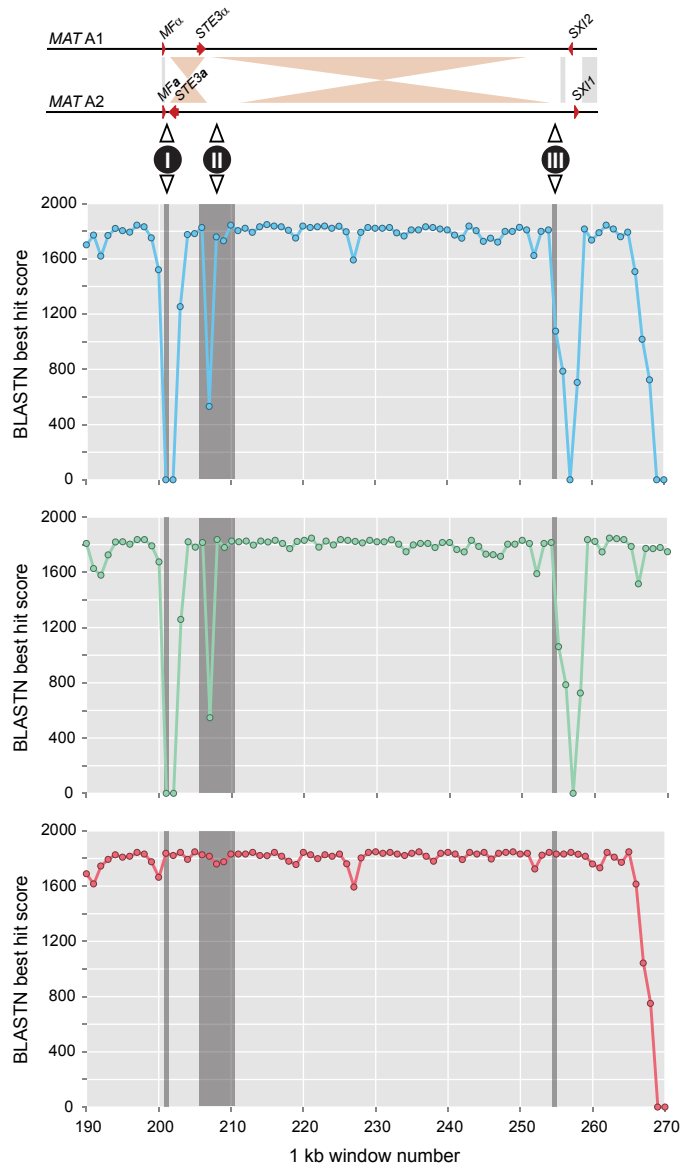

Supplement: S9 Fig — The MAT loci (region between the pheromone gene/STE3 and the corresponding HD gene) and 200 kb upstream and downstream from strains JCM1457 and UJ1 were split into fragments of 1 kb and used in BLASTN comparisons against the corresponding regions from the indicated strains. BLASTN scores for the best hits were plotted against the corresponding fragment. Higher BLASTN scores indicate higher sequence similarity, more diverse regions have lower scores. A. Overview for the three pairwise comparisons. The region shaded in yellow is the core MAT region (between the P/R genes and the corresponding HD gene). B. Detailed view of pairwise comparisons spanning sliding windows #190–270 that encompass the MAT region. Grey shading indicates the windows that harbor potential inversion breakpoints for the two inverted regions that are indicated in a schematic view above. The three regions (I-III) have a lower degree of similarity in comparisons between the A1 and A2 strains than in comparison between the two A2 strains (UJ1 and JCM1457). (PDF) [file pgen.1008365.s009.pdf]

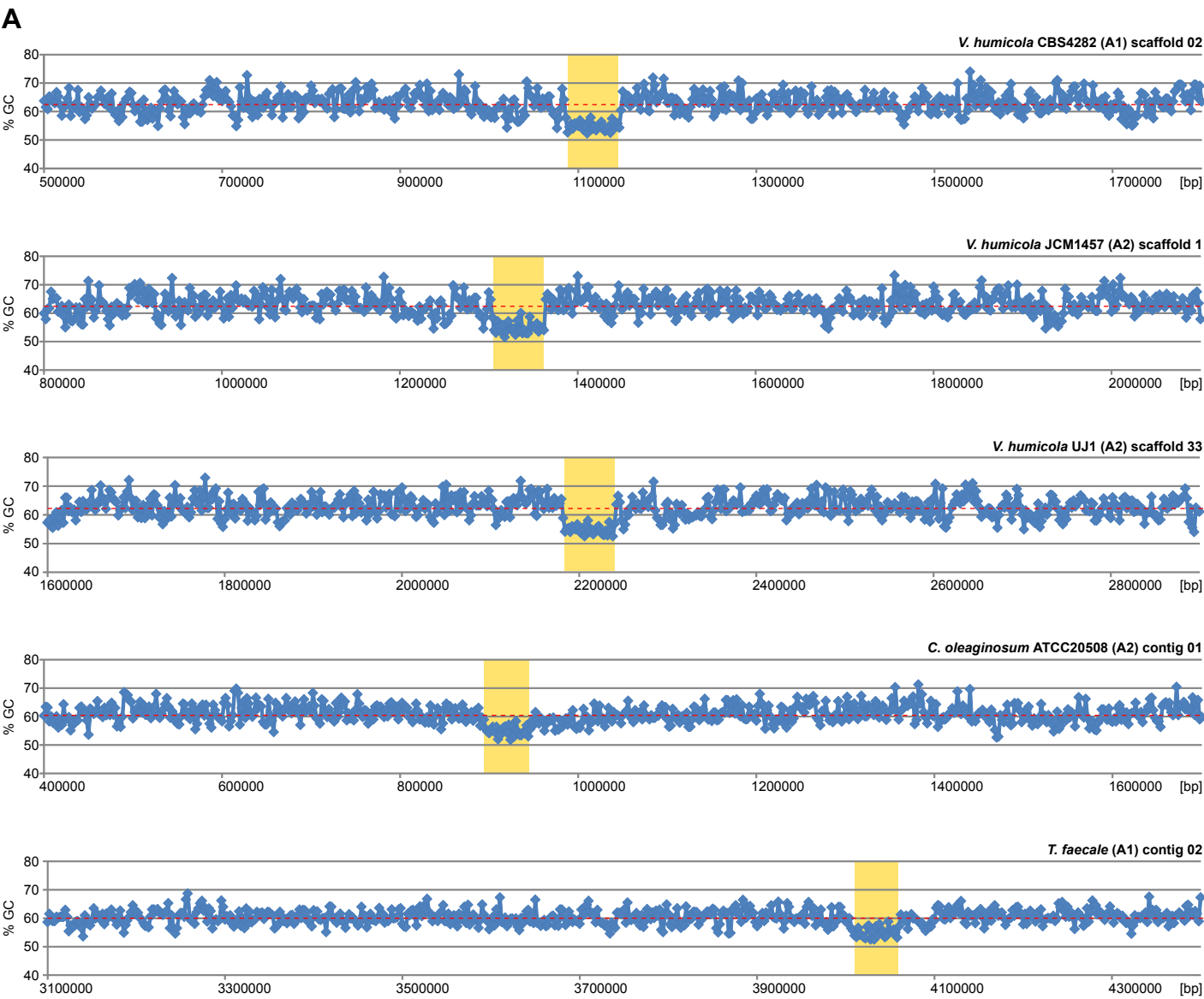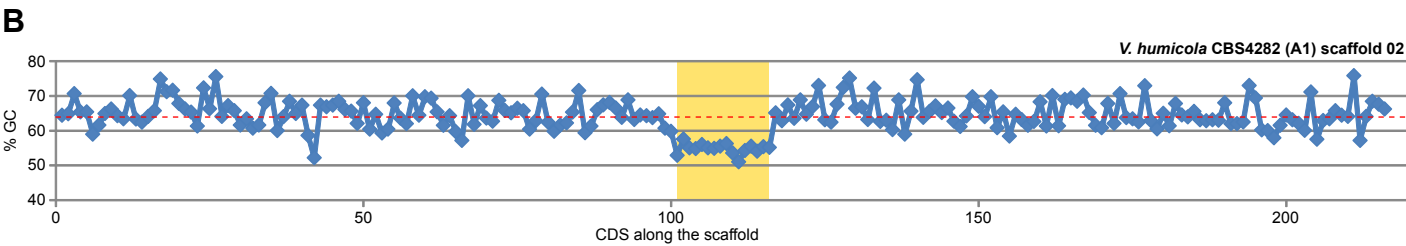

Supplement: S11 Fig — A. GC content in percent is plotted against the scaffold coordinates for the MAT-containing scaffolds. GC content was determined in windows of 2000 bp along each scaffold. The core MAT regions (between P/R and the SXI genes) are shaded in yellow for each scaffold. The average genomic GC content for each strain is indicated by a dashed red line. GC content in the core MAT region is lower than in the surrounding regions, which have GC contents that vary around the average GC content. B. GC content was calculated for each coding sequence (CDS) and plotted for 100 genes upstream and downstream from the core MAT region of strain V. humicola CBS4282.The average GC content for CDSs of this strain is indicated by a dashed red line, the core MAT region is shaded in yellow. The lower GC content observed in the genomic sequence of the MAT region is also observed when analyzing only CDS sequences. (PDF) [file pgen.1008365.s011.pdf]
