# Supplementary material for: Convergent evolution of linked mating-type loci in basidiomycete fungi
Source: PLoS Genet. 2019 Sep 6;15(9):e1008365. doi: 10.1371/journal.pgen.1008365 (PMC6730849; doi:10.1371/journal.pgen.1008365)
Supplement: S3 Fig — A conserved proline residue that is present in the α group proteins is indicated in red. It is not present in the a-specific Ste3 proteins from Tremellomycetes. (PDF) [file pgen.1008365.s003.pdf]

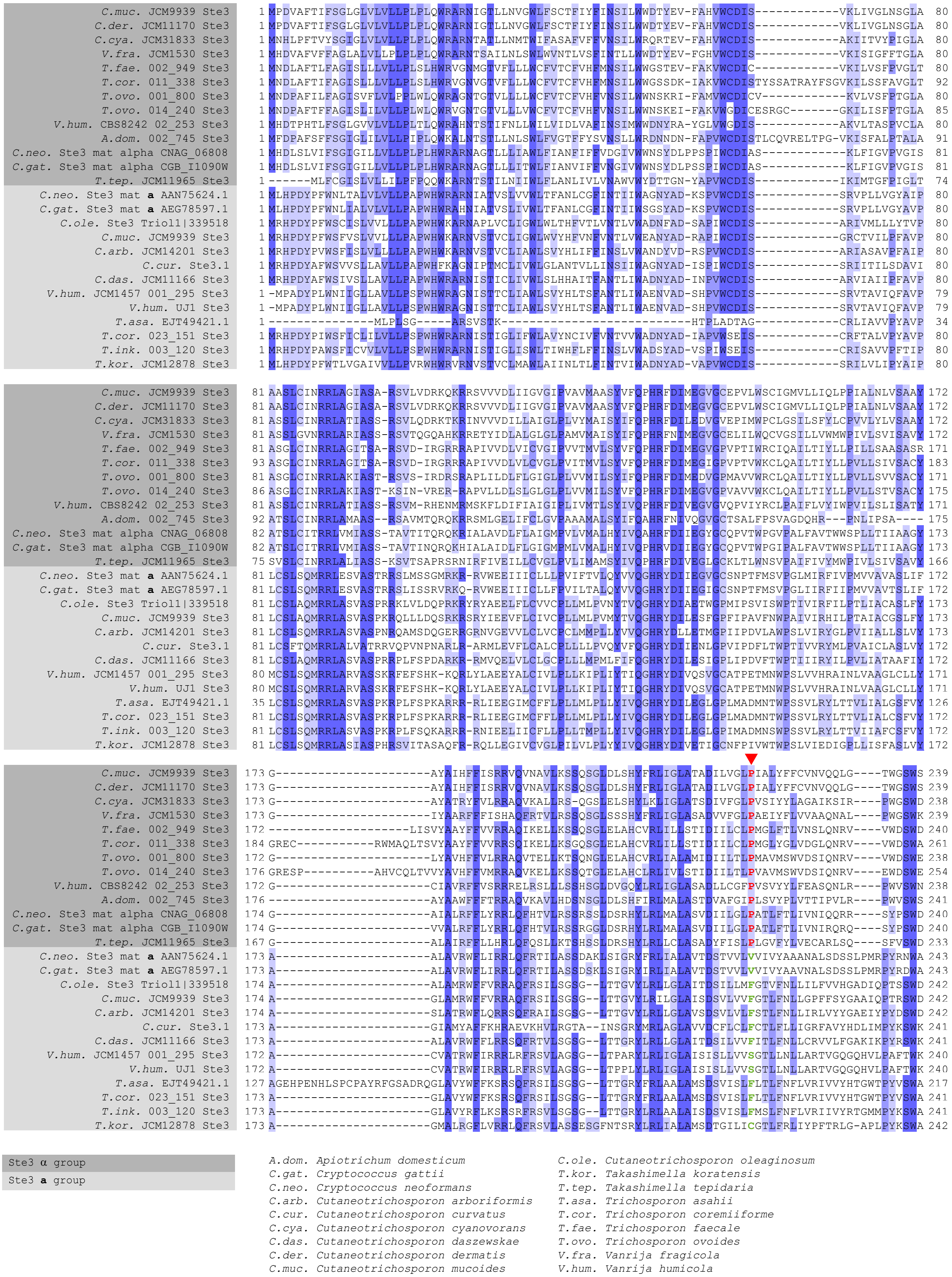
Ste3 α group
Ste3 **a** group
A.dom. Apiotrichum domesticum
C.gat. Cryptococcus gattii
C.neo. Cryptococcus neoformans
C.arb. Cutaneotrichosporon arboriformis
C.cur. Cutaneotrichosporon curvatus
C.cya. Cutaneotrichosporon cyanovorans
C.das. Cutaneotrichosporon daszewskae
C.der. Cutaneotrichosporon dermatis
C.muc. Cutaneotrichosporon mucoides
C.ole. Cutaneotrichosporon oleaginosum
T.kor. Takashimella koratensis
T.tep. Takashimella tepidaria
T.asa. Trichosporon asahii
T.cor. Trichosporon coremiiforme
T.fae. Trichosporon faecale
T.ovo. Trichosporon ovoides
V.fra. Vanrija fragicola
V.hum. Vanrija humicola
