# Supplementary material for: Convergent evolution of linked mating-type loci in basidiomycete fungi
Source: PLoS Genet. 2019 Sep 6;15(9):e1008365. doi: 10.1371/journal.pgen.1008365 (PMC6730849; doi:10.1371/journal.pgen.1008365)
Supplement: S10 Fig — Linear synteny comparison along the MAT-containing scaffolds was generated with Easyfig [131] using a minimum length of 500 bp for BLASTN hits to be drawn. (PDF) [file pgen.1008365.s010.pdf]

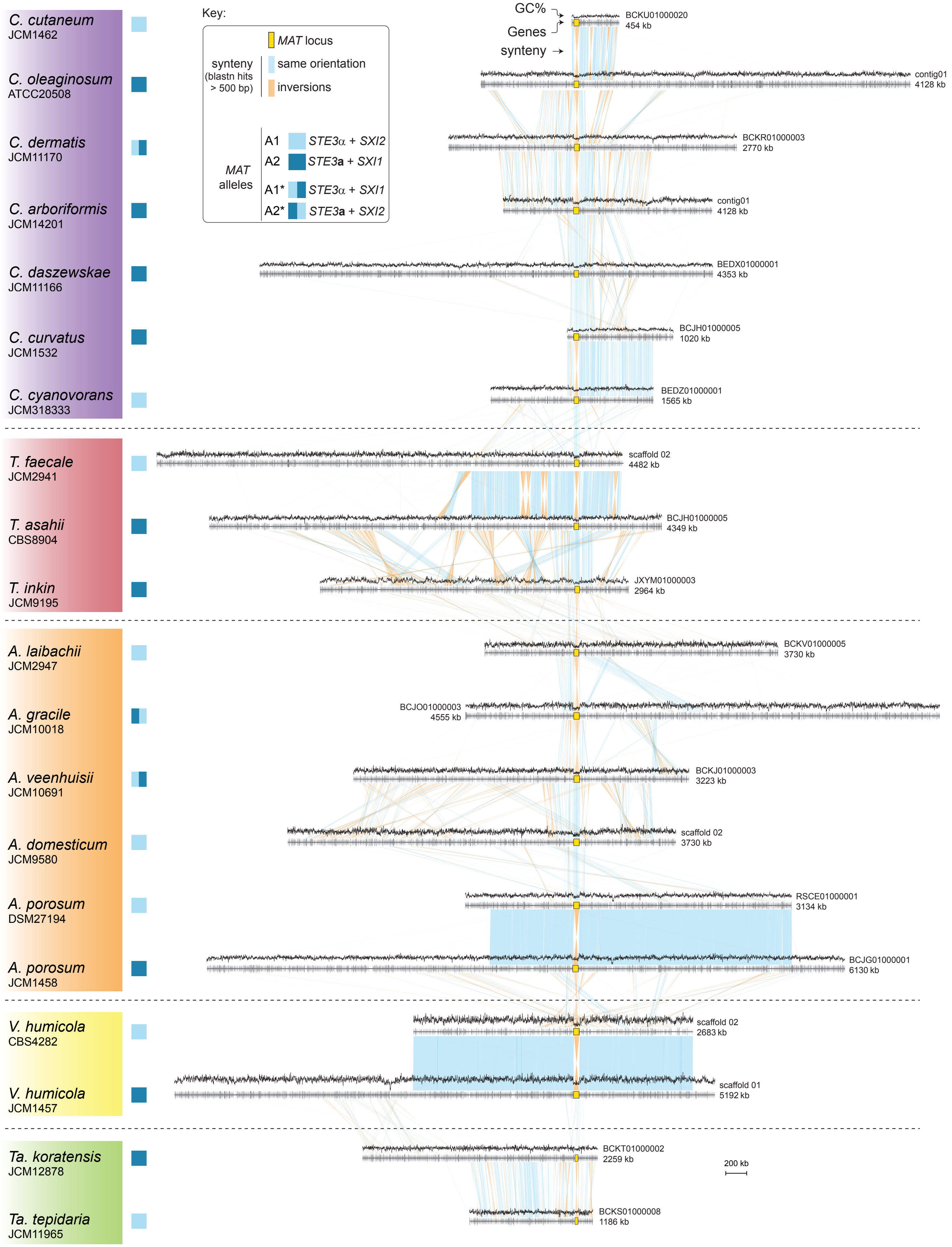

C. cutaneum JCM1462
C. oleaginosum ATCC20508
C. dermatis JCM11170
C. arboriformis JCM14201
C. daszewskae JCM11166
C. curvatus JCM1532
C. cyanovorans JCM318333
T. faecale JCM2941
T. asahii CBS8904
T. inkin JCM9195
A. laibachii JCM2947
A. gracile JCM10018
A. veenhuisii JCM10691
A. domesticum JCM9580
A. porosum DSM27194
A. porosum JCM1458
V. humicola CBS4282
V. humicola JCM1457
Ta. koratensis JCM12878
Ta. tepidaria JCM11965
Key:
MAT locus
synteny (blastn hits > 500 bp)
same orientation
inversions
MAT alleles
A1 STE3α + SXI2
A2 STE3a + SXI1
A1* STE3α + SXI1
A2* STE3a + SXI2
GC%
Genes
synteny
BCKU01000020 454 kb
contig01 4128 kb
BCKR01000003 2770 kb
contig01 4128 kb
BEDX01000001 4353 kb
BCJH01000005 1020 kb
BEDZ01000001 1565 kb
scaffold 02 4482 kb
BCJH01000005 4349 kb
JXYM01000003 2964 kb
BCKV01000005 3730 kb
BCJO01000003 4555 kb
BCKJ01000003 3223 kb
scaffold 02 3730 kb
RSCE01000001 3134 kb
BCJG01000001 6130 kb
scaffold 02 2683 kb
scaffold 01 5192 kb
BCKT01000002 2259 kb
200 kb
BCKS01000008 1186 kb
