# Supplementary material for: Convergent evolution of linked mating-type loci in basidiomycete fungi
Source: PLoS Genet. 2019 Sep 6;15(9):e1008365. doi: 10.1371/journal.pgen.1008365 (PMC6730849; doi:10.1371/journal.pgen.1008365)
Supplement: S3 Table — (PDF) [file pgen.1008365.s014.pdf]

S3 Table. Oligonucleotides used in this study.

Name	Sequence	Remarks
Vh_myo2_1	tcttctggctctcgaatgtacagg	*V. humicola* JCM1457 within *MYO2*
Vh_myo2_2	cagctgatcactcttatcttctgg	*V. humicola* JCM1457 within *MYO2*
Vh_sxi1_1	atgacggcgcctgtaagtactgc	*V. humicola* JCM1457 within *SXI1*
Vh_sxi1_2	cgaaggtggtaatgagcatcttcc	*V. humicola* JCM1457 within *SXI1*
Vh_ste3_1	gtggaacattatcggcttgcttgc	*V. humicola* JCM1457 within *STE3*
Vh_ste3_2	tcagggaactgcgagataagatcg	*V. humicola* JCM1457 within *STE3*
Vh_rpl22_1	gctcaggtacttcaaggttgacc	*V. humicola* JCM1457 within *RPL22*
Vh_01_283_1	tcggctaggaaagtgtgaagtgc	*V. humicola* JCM1457 within *001_283*
