# Supplementary material for: Convergent evolution of linked mating-type loci in basidiomycete fungi
Source: PLoS Genet. 2019 Sep 6;15(9):e1008365. doi: 10.1371/journal.pgen.1008365 (PMC6730849; doi:10.1371/journal.pgen.1008365)
Supplement: S1 Text — (PDF) [file pgen.1008365.s015.pdf]

S1 Text. Pheromone genes of *Tremellomycetes*.

One difference between the *Trichosporonales* and several *Tremellales MAT* loci is the presence of only one pheromone precursor gene in the analyzed *Trichosporonales MAT* loci (Fig 2), whereas more than one pheromone precursor gene is present within the *P/R* locus in the tetrapolar *Tremellales* species *C. amylolentus*, and *T. wingfieldii* as well as in the bipolar species *C. neoformans* and *Cryptococcus gattii* [1-4]. In all cases, the pheromone precursor genes can be found in the vicinity of the pheromone receptor gene *STE3*. The *STE3*α-associated pheromones are slightly shorter in both the *Trichosporonales* (26-42 amino acids) as well as the *Tremellales* (38 amino acids) than their *STE3***a**-associated counterparts (27-47 in the *Trichosporonales*, and 39-42 amino acids in the *Tremellales*) (see Figure below). The predicted *Trichosporonales* pheromones have the characteristic C-terminal CAAX motif of lipopeptide pheromones, where A is an aliphatic amino acid [5]. However, the *Trichosporonales* pheromones have a shorter N-terminus compared to the *Tremellales* pheromones, except for those from the *Vanrija* species, *C. curvatus* and *C. cyanovorans* (see Figure below).

References

1. Findley K, Sun S, Fraser JA, Hsueh YP, Averette AF, Li W, et al. Discovery of a modified tetrapolar sexual cycle in *Cryptococcus amylolentus* and the evolution of *MAT* in the *Cryptococcus* species complex. PLoS Genet. 2012;8:e1002528.
2. Metin B, Findley K, Heitman J. The mating type locus (*MAT*) and sexual reproduction of *Cryptococcus heveanensis*: insights into the evolution of sex and sex-determining chromosomal regions in fungi. PLoS Genet. 2010;6:e1000961.
3. Sun S, Yadav V, Billmyre RB, Cuomo CA, Nowrousian M, Wang L, et al. Fungal genome and mating system transitions facilitated by chromosomal translocations involving intercentromeric recombination. PLoS Biol. 2017;15:e2002527.
4. Fraser JA, Diezmann S, Subaran RL, Allen A, Lengeler KB, Dietrich FS, et al. Convergent evolution of chromosomal sex-determining regions in the animal and fungal kingdoms. PLoS Biol. 2004;2:e384.
5. Kües U, James TY, Heitman J. Mating type in basidiomycetes: unipolar, bipolar, and tetrapolar patterns of sexuality. In: Pöggeler S, Wöstemeyer J, editors. The Mycota XIV Evolution of fungi and fungal-like organisms. Berlin, Heidelberg: Springer; 2011. p. 97-160.

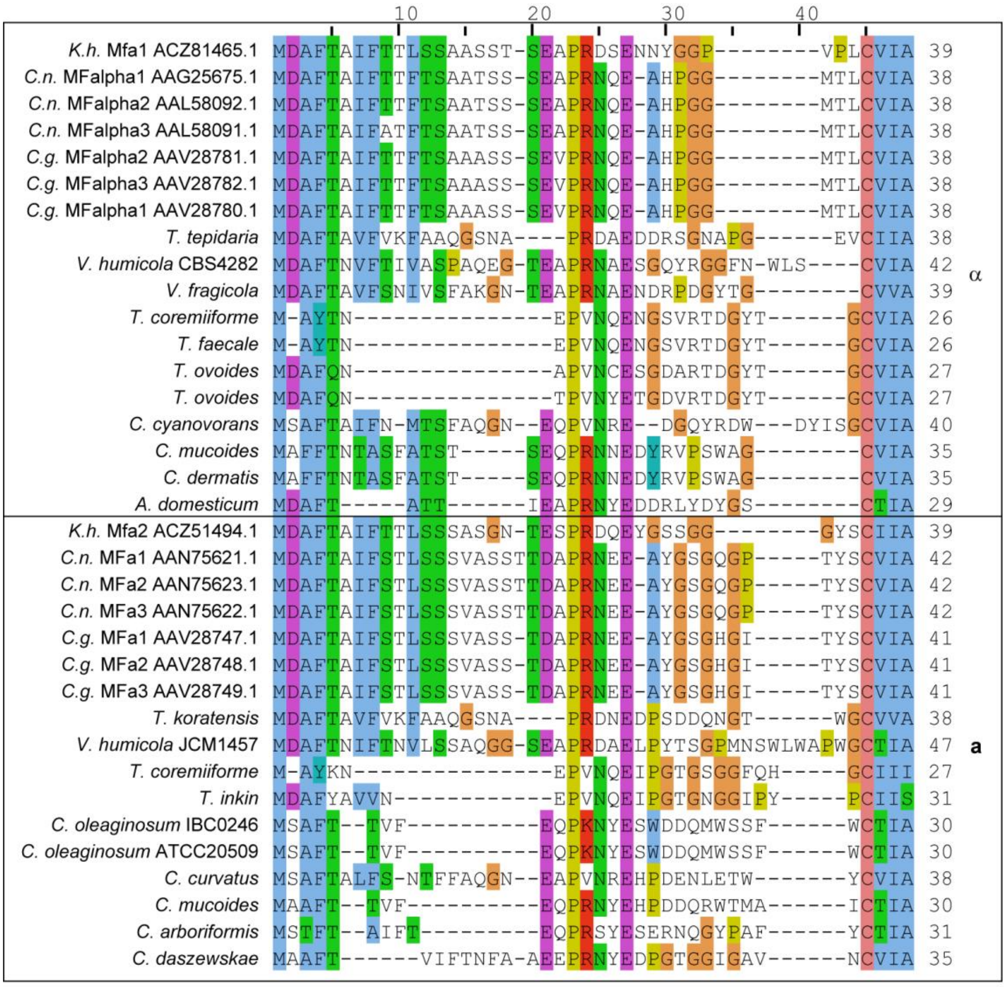

Figure for Text S1. Multiple alignment of (putative) pheromones from *Tremellomycetes*. The pheromones associated with the *STE3*α receptor gene are given on top, pheromones associated with the *STE3***a** receptor gene below. For *Tremellales* sequences, accession numbers are given after the species abbreviations (*C.n.*, *Cryptococcus neoformans*; *C.g.*, *Cryptococcus gattii*; *K.h.*, *Kwoniella heveanensis*), *Trichosporonales* sequences were identified in this study.
