# Supplementary material for: Convergent evolution of linked mating-type loci in basidiomycete fungi
Source: PLoS Genet. 2019 Sep 6;15(9):e1008365. doi: 10.1371/journal.pgen.1008365 (PMC6730849; doi:10.1371/journal.pgen.1008365)
Supplement: S2 Text — (PDF) [file pgen.1008365.s016.pdf]

S2 Text. *MAT* loci in hybrid species

Among the sequenced *Trichosporonales* genomes are three genomes derived from the hybrid species *T. coremiiforme*, *T. ovoides*, and *C. mucoides*. These species are most likely the result of prior inter-species hybridizations between closely related *Trichosporon* or *Cutaneotrichosporon* species that occurred during the evolution of the *Trichosporonales* lineage [1, 2]. Each hybrid genome has approximately twice the size of the non-hybrid genomes and still contains homeologs for the majority of genes [1, 2]. This includes the *MAT* locus, and thus, there are two *MAT* loci, each with fused *HD* and *P/R* loci, present in each genome originating from the two different species that underwent hybridization (Fig S6). The analyzed genome of the hybrid species *C. mucoides* carries both the A1* as well as the A2* allele (Fig S6B). Its closest haploid relative is *C. dermatis* [2], which carries the A1* allele, making it likely that any recombination leading to the A1* and A2* alleles occurred in the ancestor of the lineage leading to *C. mucoides* and *C. dermatis*, because the other investigated *Cutaneotrichosporon* strains from different species carry the A1 or A2 alleles. An alternative hypothesis could be that the A1* and A2* alleles are the products of an independent loss of one of the two *HD* genes.

Hybridization occurred also in the *Trichosporon* lineage [1], and the two hybrid species *Trichosporon coremiiforme* and *Trichosporon ovoides* also carry two *MAT* loci (each a fusion of one *HD* and one *P/R* locus) per genome. The analyzed *T. coremiiforme* genome contains an A1 and an A2 allele, while the analyzed *T. ovoides* genome carries two A1 alleles (Fig S6A). Sequence similarity between the two alleles present in each of the three hybrid strains, which represent three different hybrid species, is much lower than between the two alleles present in the *V. humicola* strains CBS4282 and JCM1457 (see Figure below). Thus, it is likely that the two alleles in each of the hybrid strains originated from the different parental species forming the hybrid. One question is why the presence of two different *MAT* alleles, and consequently an *SXI1* and *SXI2* gene, in *C. mucoides* and *T. coremiiforme* does not initiate sexual development including meiosis. One possibility is that the HD proteins from different species are not able to form a functional heterodimer. Alternatively, genetic events, e.g. loss of homeologous genes, might have occurred following fusion that prevent self-fertile development. Another interesting point is the finding of two A1 alleles in the analyzed *T. ovoides* genome (Fig S6A), making it unlikely that the fusion of the parental strains was part of the cell fusion that occurs during mating of strains carrying different alleles during sexual reproduction. It is possible that this was a fusion of vegetative cells, or part of a "unisexual reproduction" (same-sex mating) life cycle in the parental species. Another possibility could be mating between a diploid A1/A2 hybrid with an A1 haploid, or between two A1/A2 hybrids. In these cases, random assortments will lead to progeny with two same *MAT* alleles.

References

1. Sriswasdi S, Takashima M, Manabe R, Ohkuma M, Sugita T, Iwasaki W. Global deceleration of gene evolution following recent genome hybridizations in fungi. Genome Res. 2016;26:1081-90.
2. Takashima M, Sriswasdi S, Manabe RI, Ohkuma M, Sugita T, Iwasaki W. A *Trichosporonales* genome tree based on 27 haploid and three evolutionary conserved 'natural' hybrid genomes. Yeast. 2018;35:99-111.

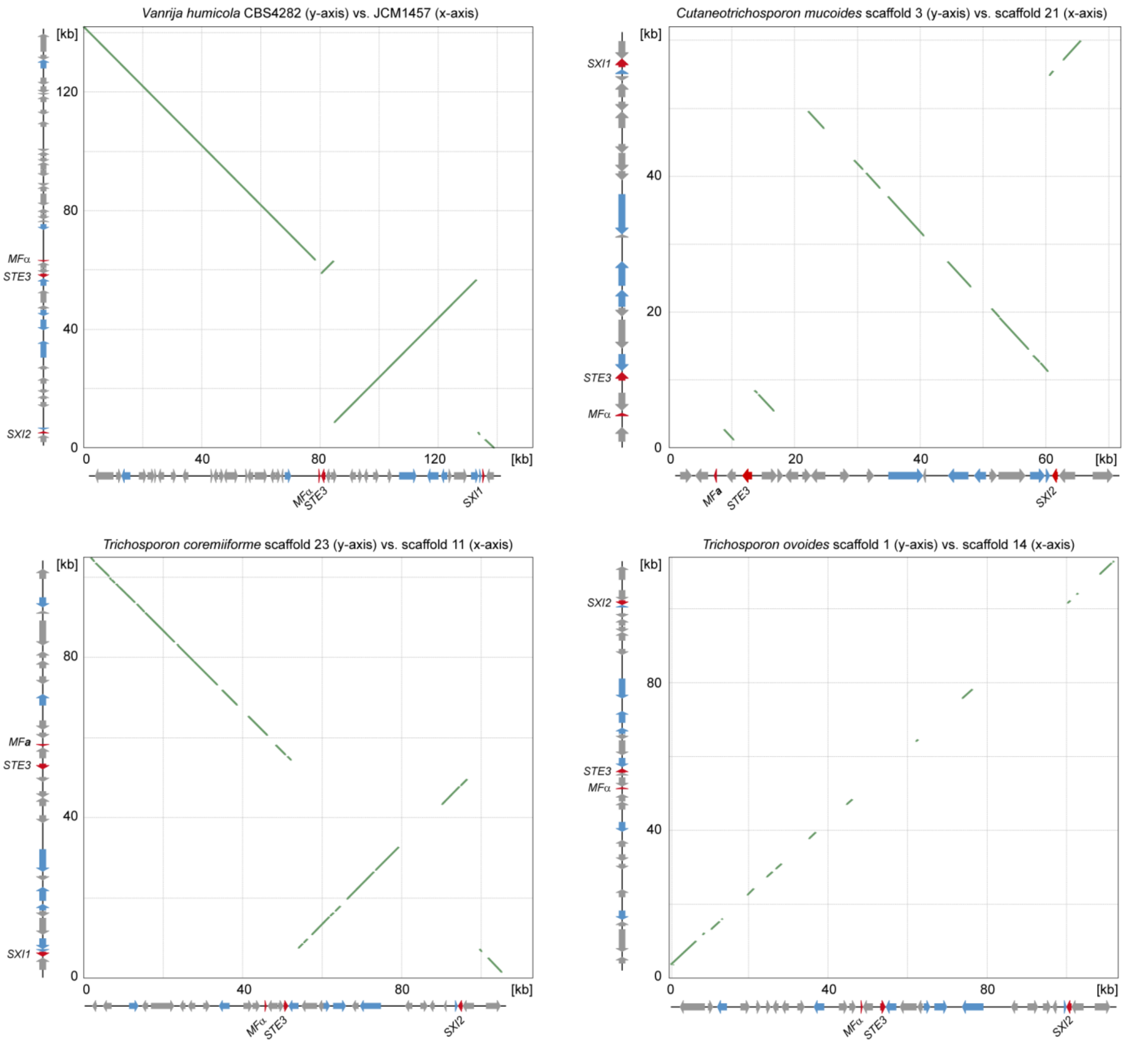

Figure for Text S2. Dot plots of sequence comparisons between *MAT* loci. Nucleic acid sequences of *MAT* loci for two *V. humicola* strains carrying the A1 and A2 alleles, and between the two alleles present in the hybrid strains *C. mucoides*, *T. coremiiforme*, and *T. ovoides* were performed with the nucmer algorithm from the MUMmer package. Dot plots were generated with gnuplot. The highest degree of similarity is observed between the two V. humicola strains, whereas the two alleles within each of the three hybrid strains are less similar to each other confirming the inter-species hybridization origin of the strains. The inversion of the region between the *STE3* and *SXI* genes can be seen in comparisons of different alleles (in the *V. humicola*, *C. mucoides*, and *T. coremiiforme* comparisons).
